# Comprehensive analysis of differential immunocyte infiltration and the potential ceRNA networks during epicardial adipose tissue development in congenital heart disease

**DOI:** 10.1186/s12967-020-02279-y

**Published:** 2020-03-02

**Authors:** Li Ma, Wanting Shi, Xun Ma, Minghui Zou, Weidan Chen, Wenlei Li, Rongjun Zou, Xinxin Chen

**Affiliations:** 1 Department of Cardiac Surgery, Guangzhou Women and Children’s Medical Center, Guangzhou Medical University, Guangzhou, 510623 Guangdong China; 2 Department of Paediatrics, Guangzhou Women and Children’s Medical Center, Guangzhou Medical University, Guangzhou, 510623 Guangdong China

**Keywords:** Congenital heart disease, Competing endogenous RNA network, Immunocyte infiltration, Bioinformatics analysis

## Abstract

**Background:**

To detect the development, function and therapeutic potential of epicardial adipose tissue (EAT); analyze a related gene expression dataset, including data from neonates, infants, and children with congenital heart disease (CHD); compare the data to identify the codifferentially expressed (DE) mRNAs and lncRNAs and the corresponding miRNAs; generate a potential competitive endogenous RNA (ceRNA) network; and assess the involvement of immunocyte infiltration in the development of the EAT.

**Methods:**

Multiple algorithms for linear models for microarray data algorithms (LIMMA), CIBERSORT, gene-set enrichment analysis (GSEA), and gene set variation analysis (GSVA) were used. The miRcode, miRDB, miRTarBase, and TargetScan database were used to construct the ceRNA network. The Gene Ontology (GO) and Kyoto Encyclopedia of Genes and Genomes (KEGG) pathway enrichment analyses of the DE mRNAs were performed.

**Results:**

Thirteen co-DE mRNAs and 47 co-DE lncRNAs were subsequently identified. The related categories included negative regulation of myoblast differentiation, regulation of ion transmembrane transport, and heart development, which were primarily identified for further pathway enrichment analysis. Additionally, the hub ceRNA network in EAT development involving *MIR210HG*, hsa-miR-449c-5p, and *CACNA2D4* was generated and shown to target monocyte infiltration.

**Conclusion:**

These findings suggest that the pathways of myoblast differentiation and ion transmembrane transport may be potential hub pathways involved in EAT development in CHD patients. In addition, the network includes monocytes, *MIR210HG*, and *CACNA2D4*, which were shown to target the RIG-I-like receptor signaling pathway and PPAR signaling pathway, indicating that these factors may be novel regulators and therapeutic targets in EAT development.

## Background

Epicardial adipose tissue (EAT), located around the heart and coronary vessels, is a complex metabolic and endocrine organ that has been proposed to have dual roles in various physiological or pathophysiological processes. EAT is known to have a protective role as a mechanical buffer and a rapid source of free fatty acids (FFAs) in the myocardium and vascular arteries [1]. There are key metabolic differences between the EAT and other adipose tissue depots. Importantly, a systematic review from Iacobellis’s group revealed that the rate of fatty acid synthesis, release, and breakdown was significantly higher in EAT than other visceral fats, and EAT exhibited lower glucose utilization as well [2]. Additionally, EAT appears to be rich in saturated fatty acids and metabolically related proteins and may have protective effects on the myocardium and vascular arteries by supplying the energy substrates for use in the ischemic myocardium and against elevated levels of FFAs in the myocardium and vascular damage to the arteries [3]. The higher lipolytic potential of EAT may also help meet the increasing myocardial energy demands, especially in the case of exercise-related metabolism or ischemic conditions [1, 4, 5]. Although fatty acids may contribute to the major energy substrates that are used to maintain cardiac metabolism and contractile function, the uptake of FFAs also leads to the impairment of mitochondrial structure and function as well as accumulation of toxic metabolites, including membrane potential disorder, Ca^2+^ overload, and cytochrome c (Cyt-C) release; these changes induce oxidative stress and disrupt metabolic pathways [6]. Anatomically, EAT shares a common blood supply with the underlying myocardium without the fascia-like structure [5]. Researchers increasingly believe that EAT contributes to pathophysiological processes related to coronary atherosclerosis, diastolic dysfunction, atrial fibrillation, and progressive ventricular remodeling, exerting exocrine and paracrine effects on the heart vasculature [4, 7, 8]. Recently, data have increasingly showed that EAT contains a high density of lymphoid clusters, and it is generally accepted to be a source of immunomodulatory and inflammatory mediators [9]. Of note, there is a mutually reinforcing relationship in the pathological processes of EAT formation and cardiac remodeling.

For adult patients, Patel et al. revealed that EAT could secrete multiple adipokines, including apelin, adiponectin, adrenomedullin, angiotensinogen, leptin, and visfatin, coupled with the potential paracrine function of regulating inflammation, immune response, coronary plaques, ventricular remodeling, and vascular smooth muscle cell growth and migration [5]. Sakamoto et al. discovered a significant association between immuno-inflammatory mediators and coronary artery calcification, which may reflect immuno-inflammation mechanisms related to the development of cardiovascular remodeling [7]. Interestingly, inflammatory cytokines, inflammatory adipokines, and M1/M2 macrophages significantly accumulate in EAT, aggravating ischemic conditions as well as the damage due to myocardial infarction [7]. Park et al. analyzed the evidence from clinical studies and clearly demonstrated that EAT thickness could predict the occurrence of metabolic syndrome and coronary atherosclerosis in an Asian population and noted that it was stronger in patients with BMI < 27 kg/m^2^ [10]. Additionally, for pediatric patients, the nutrition demand and morphology of organ development in the infant stage is critical for life. Ojha et al. found that there are unique gene expression patterns in EAT tissues in a comparison of thermogenic gene transcripts of in various developmental stages (~ 10%) [11]. Chechi et al. found that the expression of thermogenic genes significantly correlated with oxidative stress- and immune-related pathways, also presenting an important role in microenvironmental regulation within EAT tissue and local crosstalk between the cardiomyocytes and interstitial cells [12, 13].

In this scenario, EAT, an immune and endocrine organ, may function as a key inflammatory mediator for cardiovascular system development. Here, the insulin-like growth factor 1 receptor (*IGF1R*) was shown to be stimulated after myocardial damage and then promotes the differentiation of epicardial cells into adipocytes to protect cardiomyocytes and ensure the survival of cardiac progenitors [14]. In contrast, EAT serves as a secondary lymphoid organ that regulates the innate and adaptive immune responses and thus locally and systemically promotes immune cell proliferation, activates inflammatory receptors, and recruits lipid deposition across the vessel wall [9]. In this study, we performed a systematic microarray analysis to reveal the gene expression changes, inflammatory cell infiltrates, and noncoding RNA-immunocyte coregulation networks that may help us identify novel biomarkers and therapeutic targets.

## Methods

### Data sources and processes

**Fig. 1:**
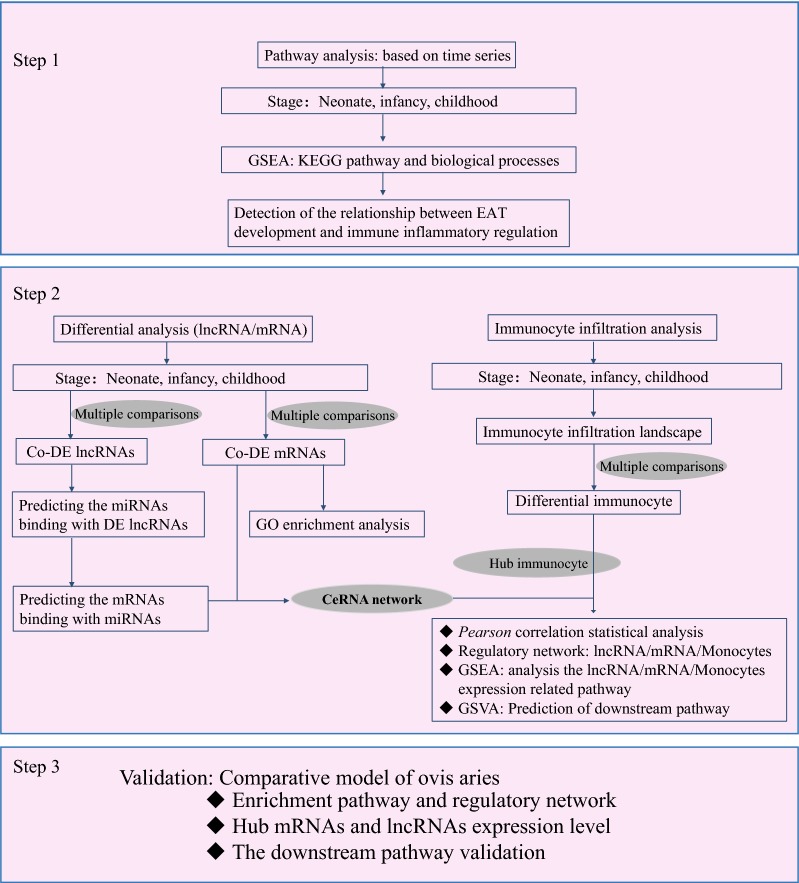
The schema of the integrative bioinformatics analyses

We performed a differential expression (DE) analysis based on “affy” package of R software that was curated by data processing principles includes expression data background correction, log2 transformation, data normalization and linear model and DE analyses. Here, the data normalization, missing value procession, and powerful DE analyses were performed by a function of the robust multi-array average (RMA), K-nearest neighbor (KNN), and linear models for microarray data algorithms (LIMMA), respectively [15]. We defined the DE threshold adjusted *p*-value, corrected by the Benjamini–Hochberg method, as less than 0.05 and |log_2_ fold-change (FC)| greater than 1.5. Herein, these genes were identified as having significantly increased (log_2_FC > 1.5) or decreased (log_2_FC < − 1.5) expression. Figure 1 illustrates the schema of the analysis flow, and the statistical analysis was performed with the R (version 3.6.1) and Perl (version 5.30.1) toolkit.

### Detection of immune infiltration subtypes

In this routine, we applied the CIBERSORT algorithm (https://cibersort.stanford.edu/) to estimate the burden of immune infiltration subtypes in the EAT development process, based on a linear support vector regression (SVR) algorithm with gene expression data with systems-level gene expression data in the R toolkit [16]. We chose the hub cell-subtypes with the parameters that were set as follows: (1) all the gene expression data were normalized by RMA algorithm; (2) the perm parameter was 1000; (3) the statistical differences between groups had *p*-value < 0.05; (4) multiple comparisons among the neonatal, infant and child groups were tested by one-way ANOVA, and the *p*-value was adjusted by the *Bonferroni* method.

### Gene set bioinformatics enrichment analysis

Gene Ontology (GO) term enrichment analyses were carried out separately for the DE genes using the MetaScape database (http://metascape.org/), which is a web-based analytics tool used for gene functional enrichment analysis [17]. Of note, the major GO terms of the DE genes in biological processes, molecular functions, and cellular components were illustrated. The parameter settings were as follows: (1) min overlap is 3; (2) the cutoff *p*-value = 0.05; (3) the min enrichment score = 1.5.

Based on the time series-related Kyoto Encyclopedia of Genes and Genomes (KEGG) pathway and biological process term enrichment, a gene-set enrichment analysis (GSEA; http://software.broadinstitute.org/gsea/index.jsp) was performed [18]. The parameters were as follows: (1) the threshold for significant enrichment was *p*-value < 0.05; (2) the permutation test number = 1000; (3) the gene profile was defined as continuous phenotypes with time series experiments for EAT development and defined as class labels in response to the expression level of hub mRNAs/immunocyte infiltration; (4) enrichment statistic was used for weighting; (5) the metric for ranking genes selected “*Pearson*” for time series analysis and defined the “Signal2Noise” for categorical analysis.

### Competing endogenous RNA (ceRNA) regulatory network construction

The ceRNA networks of lncRNAs, miRNAs, and mRNAs were predicted based on the miRcode (http://www.mircode.org/) [19], miRDB (http://mirdb.org/) [20], miRTarBase (http://mirtarbase.mbc.nctu.edu.tw/php/index.php) [21], and TargetScan (http://www.targetscan.org/mamm_31/) databases [22]. Here, the analysis was divided into several steps including: (1) calculating the differentially expressed lncRNAs based on the HUGO Gene Nomenclature Committee (HGNC) Database and NetAffx annotation of the Affymetrix HG-U133 Plus 2.0 array and DE analysis [23]; (2) predicting the miRNAs binding with DE lncRNAs based on the highly conserved microRNA family files of the miRcode database; (3) predicting the mRNAs binding with miRNAs, identified from the previous step, based on a comparison to the annotation files of the miRDB, miRTarBase, and TargetScan databases with the criterion for the matching database number ≥ 2; (4) selecting the hub mRNAs after determining the overlapping target-mRNAs and DE mRNAs.

### Identification of the downstream regulatory pathways of target genes

After grouping by the median expression value of the target gene, lncRNA, and infiltrating immunocytes, we applied the gene set enrichment analysis (GSEA) to detect the downstream pathways. The normalized enrichment score (NES) was obtained via cluster analysis based on the *Pearson* statistical method [18]. In addition, after identification of the potential regulatory pathways, the gene set variation analysis (GSVA) package, based on a nonparametric unsupervised method, was applied to explore the expression of hub genes in each sample in relation to the hub ceRNA network and candidate immunocytes in our study [24].

### Validation of candidate immunocytes, regulatory networks and hub genes

Finally, we also verified the landscape of immunocyte infiltration, regulatory networks and hub gene expression related to EAT development based on the GSE115799 dataset for *Ovis aries*. The CIBERSORT algorithm was used to compare the EAT sample immune infiltration between 1 and 4 weeks of age. Furthermore, the candidate pathway variation was assessed by the GSVA package. The mRNA and lncRNA expression values between the two groups were compared with powerful DE analyses with the LIMMA algorithm.

## Results

### Detection of hub pathways and differentially expressed genes

**Fig. 2:**
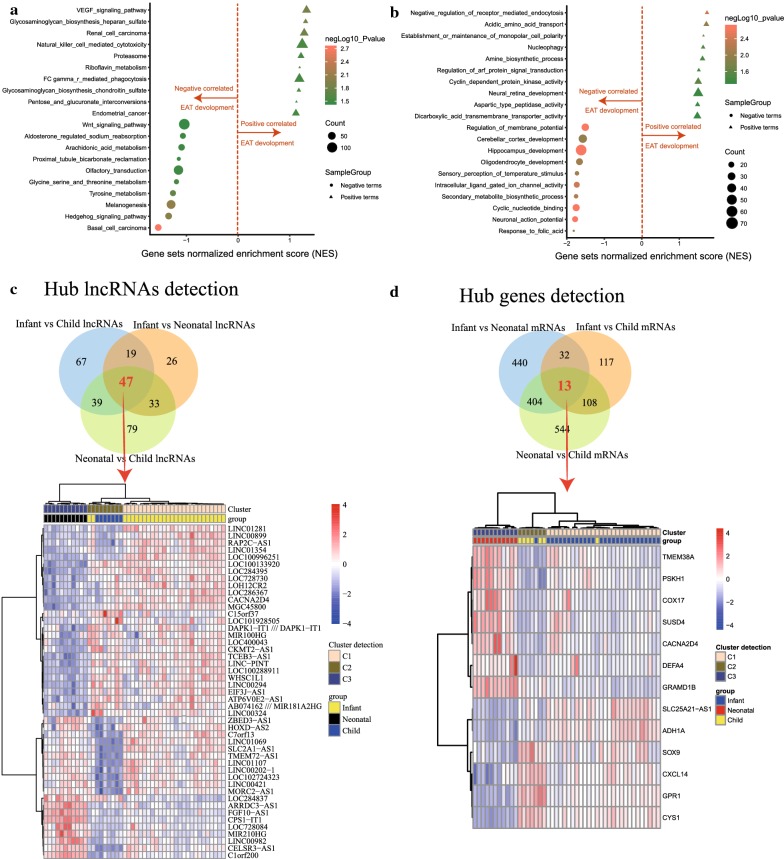
Detection of the hub pathways and differentially expressed genes for epicardial adipose tissue (EAT) development. **a**, **b** The Kyoto Encyclopedia of Genes and Genomes (KEGG) pathway (Fig. 1a) and biological process terms of Gene Ontology (GO) (Fig. 1b) were detected by gene set enrichment analysis (GSEA) based on a time series analysis. **c**, **d** The Venn diagram and heatmap plots showing the codifferentially expressed (co-DE) mRNAs and co-DE lncRNAs identified from the multigroup comparisons among the infant, neonatal, and child EAT tissues from congenital heart disease (CHD) patients

The GSEA results, based on the time series analysis, showed that the KEGG categories of basal cell carcinoma (size = 52, NES = − 1.56, *p*-value = 0.001), melanogenesis (size = 96, NES = − 1.32, *p*-value = 0.009), and hedgehog signaling pathway (size = 53, NES = − 1.36, *p*-value = 0.011) were negatively correlated with EAT development, while other categories including the VEGF signaling pathway (size = 70, NES = 1.33, *p*-value = 0.015), glycosaminoglycan biosynthesis heparan sulfate (size = 26, NES = 1.31, *p*-value = 0.015), and renal cell carcinoma (size = 69, NES = 1.28, *p*-value = 0.016) were positively associated with EAT development (Fig. 2a; Additional file 1: Table S1). The biological processes from the GO analysis include hippocampus development (size = 72, NES = − 1.65, *p*-value = 0.002), cyclic nucleotide binding (size = 34, NES = − 1.77, *p*-value = 0.002), and neuronal action potential (size = 26, NES = − 1.80, *p*-value = 0.002), which were negatively correlated with EAT development, and negative regulation of receptor mediated endocytosis (size = 17, NES = 1.74, *p*-value = 0.004), acidic amino acid transport (size = 21, NES = 1.72, *p*-value = 0.010), and establishment or maintenance of monopolar cell polarity (size = 15, NES = 1.64, *p*-value = 0.019), which were positively correlated with EAT development (Fig. 2b; Additional file 1: Table S1).

Of note, 46 EAT samples were obtained from cardiopulmonary bypass cardiac surgery of pediatric patients with congenital heart disease (CHD). Here, for the comparison among the neonate, infant, and child EAT samples, to illustrate the transcription and expression profiles in response to EAT development, we performed a powerful differential analysis across the comparison groups. The results showing that 270, 889, and 1069 DE mRNAs were detected in the comparisons of infant *vs* child, infant *vs* neonate, and neonate *vs* child, respectively (Fig. 2c; Additional file 2: Table S2). Similarly, 172, 125, and 198 DE lncRNAs were identified in the comparisons of infant *vs* child, infant *vs* neonate, and neonate *vs* child, respectively (Fig. 2d; Additional file 2: Table S2). The 13 co-DE mRNAs and 47 co-DE lncRNAs were subsequently identified. In addition, co-DE mRNAs and lncRNAs were identified to construct the heatmap. A cluster analysis based on the *Pearson* method presented a significant intergroup difference (Fig. 2c, d).

### Pathway enrichment analysis

**Fig. 3:**
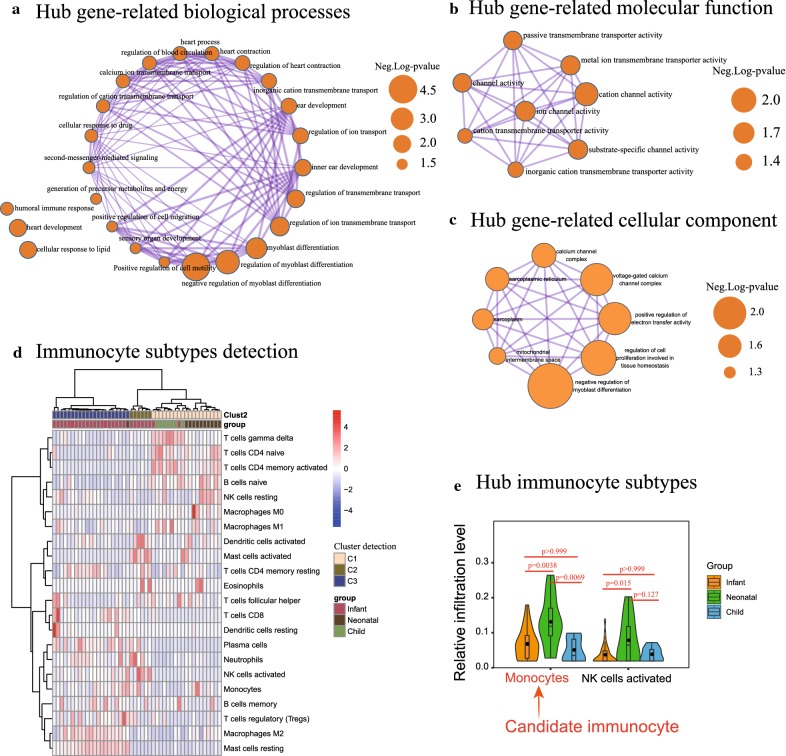
Detection of the Gene Ontology (GO) terms and immunocyte infiltration pattern. **a**–**c** The terms, including biological process (BP), molecular function (MF), and cellular composition (CC), were mapped form the MetaScape database regarding the co-DE mRNAs. The sizes of the dots representing the negative log(*p*-value). **d**, **e** The heatmap cluster of Fig. 2d illustrating the landscape of immunocyte infiltration in response to different developmental phases of EAT tissues. Figure 2e presenting the statistically significant immunocytes

Of the 13 coexpressed DE genes, the biological functions were primarily associated with negative regulation of myoblast differentiation (*p*-value = 7.96E−05), regulation of ion transmembrane transport (*p*-value = 0.002), and heart development (*p*-value = 0.003) (Fig. 3a; Additional file 3: Table S3). The molecular functions were significantly associated with cation channel activity (*p*-value = 0.012), ion channel activity (*p*-value = 0.020), and substrate-specific channel activity (*p*-value = 0.021) (Fig. 3b; Additional file 3: Table S3), as well as negative regulation of myoblast differentiation (*p*-value = 7.96E−05), regulation of cell proliferation involved in tissue homeostasis (*p*-value = 5.38E−04), and positive regulation of electron transfer activity (*p*-value = 0.001) for cellular components (Fig. 3c; Additional file 3: Table S3).

### Immunocyte subtype detection

As shown in Fig. 3d, the landscape of immunocyte infiltration in response to EAT development was detected after calculation by the CIBERSORT algorithm. Based on the statistical analysis of EAT samples at different stages, the monocytes (infant vs neonate: *p*-value = 0.0038, t = 3.45; infant vs child: *p*-value > 0.999, t = 0.799; child vs neonate: *p*-value = 0.0069, t = 3.242) and NK cells activated (infant vs neonate: *p*-value = 0.015, t = 2.956; infant vs child: *p*-value > 0.999, t = 0.0937; child vs neonate: *p*-value = 0.127, t = 2.093) were identified (Fig. 3d; Additional file 4: Table S4). As a result, the immunocytes of monocytes, as a hub immune cell fraction, were quantified from the gene expression matrix derived from the EAT samples (Fig. 3e).

### CeRNA network construction

**Fig. 4:**
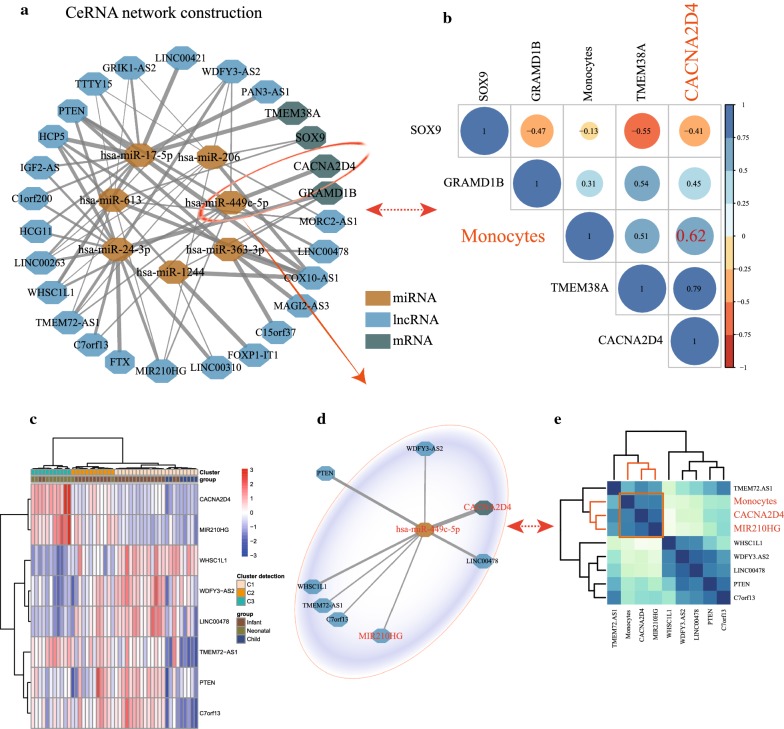
The ceRNA network construction and detection related to infiltrating immunocytes. **a** The network plot presenting a regulatory landscape based on co-DE mRNAs, co-DE lncRNAs, and targeting miRNAs. **b** Fig. 3b showing *Pearson*’s correlation coefficients for the hub mRNAs and candidate immunocytes. **c**, **d** After determination of the hub regulatory network, Fig. 3c shows a clustering analysis for hub mRNAs and lncRNAs, thus reflecting the sample discrimination ability. **e** Figure 3e demonstrates that the *MIR210HG*, *CACNA2D4*, and monocytes infiltration values were clustered

Our findings, including (1) 1742 links, were calculated based on the 47 co-DE lncRNAs and targeted miRNAs, (2) and 27 lncRNAs and 7 miRNAs were selected after removing the duplicate nodes, (3) and 4 targeted mRNAs were selected after determination of the miRNAs targeting mRNAs and co-DE mRNAs in multiple group comparisons. As a consequence, the ceRNA network including 27 lncRNAs, 7 miRNAs, and 4 mRNAs was accordingly constructed for EAT development for CHD patients (Fig. 4a; Additional file 5: Table S5).

A significant coefficient of correlation linking the infiltrating value of monocytes and the expression value of *CACNA2D4* was identified (*Pearson*’s correlation coefficient = 0.62, *p*-value = 4.32E−06) (Fig. 4b). Subsequently, a subnetwork including *CACNA2D4*, *hsa*-*miR*-*449c*-*5p*, *C7orf13*, *PTEN*, *LINC00478*, *WDFY3.AS2*, *WHSC1L1*, and *MIR210HG* was identified as a core regulatory network (Fig. 4d). The cluster analysis results of these biomarkers showed significant discrimination in response to different developmental stages of EAT (Fig. 4c). Then, to further elucidate the lncRNA–miRNA–mRNA regulatory mechanism in response to monocyte infiltration, we calculated *Pearson*’s correlation coefficient values for these biomarkers. A high-density correlation module including monocytes, *MIR210HG*, and *CACNA2D4* was generated (Fig. 4e; Additional file 6: Table S6).

### GSEA and GSVA analysis

**Fig. 5:**
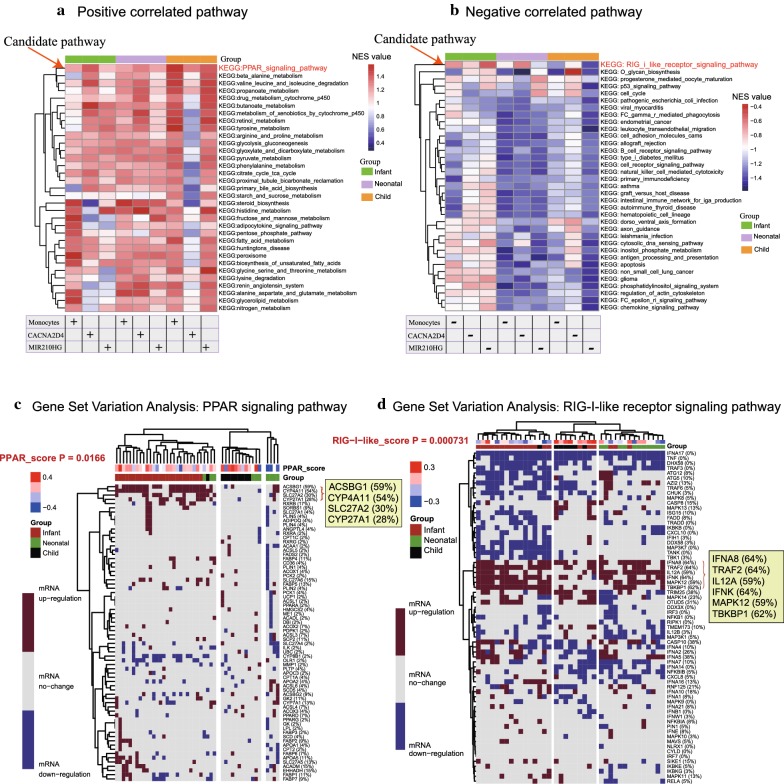
The GSEA and GSVA in response to *MIR210HG*, *CACNA2D4* gene expression, and monocyte infiltration. **a**, **b** After grouping, Fig. 4a shows the significant terms correlated with higher monocyte infiltration, higher *CACNA2D4* expression, and higher *MIR210HG* expression, while Fig. 4b presents the opposite case. **c**, **d** Fig. 4c, d demonstrate the enriched genes of the PPAR signaling pathway and RIG-I-like receptor signaling pathway

The GSEA analysis was applied to elucidate the potential regulatory pathways after grouping by the median value. The cluster analysis of the pathway NES value showed that the PPAR signaling pathway was significantly correlated with higher monocyte infiltration, higher *CACNA2D4* expression, and higher *MIR210HG* expression (Fig. 5a and Additional file 7: Table S7). In contrast, the RIG-I-like receptor signaling pathway was primarily associated with lower monocyte infiltration, lower *CACNA2D4* expression, and lower *MIR210HG* expression (Fig. 5b).

Additionally, genes showing significant variation in different stages of EAT development, including *ACSBG1* (59%), *CYP4A11* (54%), *SLC27A2* (30%), and *CYP27A1* (28%), were identified in the PPAR signaling pathway (variation score *p*-value = 0.0166; Fig. 5c). Similarly, genes such as *IFNA8* (64%), *TRAF2* (64%), *IL12A* (59%), *IFNK* (64%), *MAPK12* (59%), and *TBKBP1* (62%) were detected in the retinoic acid-inducible gene-I (RIG-I) like receptor signaling pathway (variation score *p*-value = 0.00073; Fig. 5d).

### Verification of immunocytes, regulatory maps and hub genes in an *Ovis aries* model

**Fig. 6:**
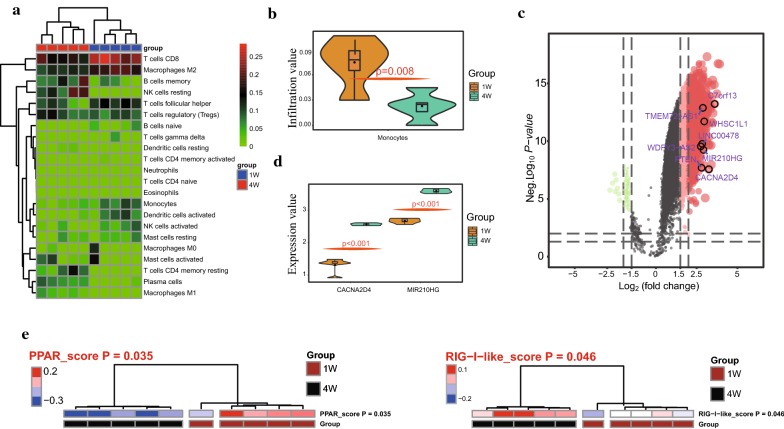
The validation of immunocyte infiltration, candidate pathways and hub genes in EAT development. **a**, **b** The immunocyte infiltration landscape at 1 and 4 weeks of age, and the infiltration level of monocytes was decreased in the 4 weeks of age group. **c** The hub genes showed differences in different developmental stages. **d** The volcano plot showing the distribution of the *CACNA2D4*, *C7orf13*, *PTEN*, *LINC00478*, *WDFY3*-*AS2*, *TMEM72*-*AS1*, *WHSC1L1*, and *MIR210HG* expression levels. **e** The clustering heatmap illustrating the PPAR signaling pathway and RIG-I like receptor signaling pathway

After analysis, the immuno-infiltration landscape is presented in Fig. 6a (Additional file 4: Table S4). Additionally, compared with that of the 7 days of age group, the infiltration level of monocytes was decreased in the 28 days of age group (Fig. 6b). The EAT transcriptomic levels of *CACNA2D4*, *C7orf13*, *PTEN*, *LINC00478*, *WDFY3*-*AS2*, *TMEM72*-*AS1*, *WHSC1L1*, and *MIR210HG* were compared between the 7 and 28 days of age groups, and the results showed that both the *CACNA2D4* and *MIR210HG* genes were upregulated in the 28 days of the age group compared with the 7 days of age group (Fig. 5c, d). Based on GSVA analysis, the PPAR signaling pathway (variation score p-value = 0.035) and the RIG-I like receptor signaling pathway (variation score p-value = 0.046) were verified and are thus considered important regulatory pathways/networks in EAT development (Fig. 6e).

## Discussion

At present, EAT is believed to be a large secretosome that has major roles in regulating the physiological and pathophysiological processes in cardiovascular disease. Furthermore, EAT exerts a potent effect on the paracrine regulation of vascular atherosclerosis susceptibility, myocardial fibrosis, and arrhythmia based on functional and anatomic proximity. In sum, EAT has been associated with thermogenesis, immune-inflammation regulation, cardiometabolic activity, and adrenergic receptor distribution and thus has been a research hot spot in recent years. For the first time, we integrated human and *O. aries* microarray-based profiles of expression data to determine the immunocyte infiltration- and the candidate immunocyte-correlated regulatory ceRNA network during the development of EAT through early life. Further extensions of the bioinformatics tools presented herein were used to identify the molecular mechanisms and genetic factors related to EAT development and immunocyte infiltration.

ceRNAs, competing for a shared pool of miRNAs, provide a complex network of interactions targeting the microRNA response elements (MREs) in protein-coding mRNAs, microRNAs, long noncoding RNAs, pseudogenic RNAs, and circular RNAs [25, 26]. Recently, numerous studies have reported widespread functions, including RNA shearing, RNA editing, RNA modification and RNA binding, in human development and disease [25, 26]. Thus, understanding the ceRNA networks linked to EAT development and immunocyte infiltration will lead to important insights into cardiac development and immunoregulation involved in the age change of EAT in CHD patients in early life.

Based on the complex bioinformatic analysis, our study demonstrated a framework to systematically characterize a regulatory map involving DE mRNAs, DE lncRNAs, immunocyte infiltration, and potentially regulated pathways. In the comparisons among the neonate, infant, and child EAT samples from CHD patients, a total of 13 co-DE mRNAs and 47 co-DE lncRNAs were identified as core regulators that are primarily involved in the pathways of myoblast differentiation, ion transmembrane transport, and heart development. We subsequently constructed a ceRNA regulatory network for these co-DE mRNAs and co-DE lncRNAs as presented above. As a result, *MIR210HG* was identified as an important competing endogenous RNA of hsa-miR-449c-5p to regulate the *CACNA2D4* expression level and thus regulate monocyte infiltration during the EAT development of CHD patients in early life. The PPAR signaling pathway was positively correlated with *MIR210HG*, *CACNA2D4* expression, and monocyte infiltration, while the RIG-I like receptor signaling pathway was inversely correlated.

Functionally, *CACNA2D4* encodes a protein of the voltage-dependent calcium channel complex that serves as a mediator leading to the influx of calcium ions through the cytomembrane. Based on the PathCards database (https://pathcards.genecards.org/) [27], we found that *CACNA2D4* may be correlated with the pathways of T cell receptor signaling and dilated cardiomyopathy (DCM). Weeke et al. showed that the calcium channel subunit genes *CACNB2* and *CACNA2D4* were significantly correlated with familial atrial fibrillation [28]. While the regulation of cardiac rhythm has been studied, the exact mechanism has not been fully elucidated [28]. The long noncoding RNA-*MIR210HG*, as reported by recent studies, may function as an oncogenic regulator that promotes tumor metastasis, proliferation, and invasion in breast cancer, glioma, hepatocellular carcinoma, and non-small cell lung cancer [29–34]. In addition, Voellenkle’s group and Lin’s group demonstrated that *MIR210HG* may be involved in the pathway of hypoxic and inflammatory stress of endothelial cells [35, 36]. Hsa-miR-449c-5p serves as an important mediator involved in brain development, gastric carcinoma growth, and liver cancer cell migration via extracellular signal and intracellular pathways that target the post-transcriptional regulation of genes [37–39]. Xu et al. found that the overexpression of hsa-miR-449c-5p may inhibit the osteogenic differentiation of valve interstitial cells via targeting Smad4 expression in calcific aortic valve disease [40].

The PPARs, including PPARα, PPARβ/δ, and PPARγ, were reported to regulate adipogenesis, lipid metabolism, and immunoinflammatory responses, especially in the maintenance of metabolic homeostasis [41]. Interestingly, recent findings have shown that PPARs, particularly PPARγ, are highly expressed in white adipose tissue (WAT) and BAT and mediate adipogenesis by regulated lipid oxidation, triglyceride accumulation, and glucose utilization under physiological and pathological conditions [41]. As the master regulator in adipocyte differentiation, PPARs also participate in angiogenesis, immune microenvironment construction and energy metabolism in adipose tissue [41–43]. However, these processes of energy metabolism and construction of the immune microenvironment could be induced by EAT development and metabolic dysregulation due to disturbances in oxidative stress, ion transport, mitochondrial oxidative phosphorylation, and myocardial energy metabolism [44, 45]. Wang et al. demonstrated that RIG-I may be a direct target and contribute to regulating the balance between the anti-inflammatory and proinflammatory responses and glucose homeostasis during the process of insulin resistance (IR), which is consistent with immune tolerance regulation [46]. In addition, the activation of the RIG-I like receptor signaling pathway may potentially link inflammation and cardiomyocyte apoptosis [47]. Renovato-Martins et al. demonstrate that monocyte infiltration into adipose tissue is a signature of organ early development, while macrophage infiltration is a hallmark of the inflammatory response in the adipose tissues of obesity patients [48]. Similarly, Wolf’s group found that the feedback loop between monocytes and macrophages not only provides immunological defense but also contributes to maintaining the function of EAT, which involves thermogenesis, metabolic balance, and local sympathetic nerve distribution [49]. Pirzgalska et al. demonstrated that monocytes-macrophages are intimately linked to the browning of white adipose tissue (WAT) and thermogenesis mediated by norepinephrine (NE) [50].

## Conclusion

Related studies have proved that EAT development was regulated by the immune-inflammatory microenvironment and that the genetic regulation was complicated. In our study, these results further revealed the potential effects and research value of myoblast differentiation and ion transmembrane transport in EAT development during early childhood, and thus, these related DE genes were further assessed in the follow-up study. Additionally, a network including monocytes, *MIR210HG*, hsa-miR-449c-5p, and *CACNA2D4* was detected in EAT development and may serve as a primary regulator controlling thermogenesis and immunoinflammatory response. These results might be used in novel strategies to develop effective therapies targeting myocardial energy supply and development in CHD patients. Our findings could further elucidate the molecular mechanisms involved in EAT, thereby enabling the exploration of EAT development and CHD pathophysiology from a new perspective, which may help improve the current therapies.

## Supplementary information

**Additional file 1: Table S1.** The epicardial adipose tissue (EAT) development was correlated with pathways based on time series-related GSEA.

**Additional file 2: Table S2.** The differentially expressed mRNAs and lncRNAs were identified in multiple comparisons among the EAT samples at different developmental stages.

**Additional file 3: Table S3.** The Gene Ontology enrichment terms for codifferentially expressed mRNAs among the neonatal, infant and child EAT samples.

**Additional file 4: Table S4.** The immunocyte infiltration detection of the GSE82155 and GSE115799 datasets.

**Additional file 5: Table S5.** The competing endogenous RNA (ceRNA) regulatory network construction.

**Additional file 6: Table S6.***Pearson*’s correlation coefficient values of hub miRNAs and monocyte infiltration level.

**Additional file 7: Table S7.** The gene set enrichment analysis after grouping by the monocyte infiltration level, *CACNA2D4* and *MIR210HG* expression value.

## Data Availability

The Gene Expression Omnibus [51] (GEO; https://www.ncbi.nlm.nih.gov/geo/) gene expression profile GSE82155, which examined changes in response to the age change of EAT for CHD patients in early life, including 11 neonate (6 to 24 days old), 28 infant (40 days to 1 year old), and 7 child (2 to 7 years old) EAT samples, was used to perform the DE analysis and gene to gene interactions by applying systems biology molecular analytical approaches. This study was validated by assessing the transcriptomic changes during *Ovis aries* early development, which includes 5 depots at 7 days and 5 depots at 28 days of age in the GSE115799 dataset. The analysis scripts can be accessed from the Github web site at https://github.com/RogerZou0108/EAT-Bio-analysis.

## Abbreviations

EAT: Epicardial adipose tissue
CHD: Congenital heart disease
DE: Differentially expressed
GO: Gene Ontology
KEGG: Kyoto Encyclopedia of Genes and Genomes
Cyt-C: Cytochrome c
GEO: Gene Expression Omnibus
RMA: Robust multi-array average
KNN: K-nearest neighbor
LIMMA: Linear models for microarray data algorithms
FC: Fold changes
FDR: False discovery rate
SVR: Support vector regression
ceRNAs: Competing endogenous RNAs
HGNC: HUGO Gene Nomenclature Committee
GSEA: Gene Set Enrichment Analysis
NES: Normalized enrichment score
GSVA: Gene Set Variation Analysis
MREs: microRNA response elements
DCM: Dilated cardiomyopathy
IR: Insulin resistance
WAT: White adipose tissue
NE: Norepinephrine

## References

[1] Gibb AA, Epstein PN, Uchida S, Zheng Y, McNally LA, Obal D, Katragadda K, Trainor P, Conklin DJ, Brittian KR (2017). Exercise-induced changes in glucose metabolism promote physiological cardiac growth. Circulation.

[2] Iacobellis G, Sharma AM (2007). Epicardial adipose tissue as new cardio-metabolic risk marker and potential therapeutic target in the metabolic syndrome. Curr Pharm Des.

[3] Iacobellis G, Malavazos AE, Corsi MM (2011). Epicardial fat: from the biomolecular aspects to the clinical practice. Int J Biochem Cell Biol.

[4] Iacobellis G (2015). Local and systemic effects of the multifaceted epicardial adipose tissue depot. Nat Rev Endocrinol.

[5] Patel VB, Shah S, Verma S, Oudit GY (2017). Epicardial adipose tissue as a metabolic transducer: role in heart failure and coronary artery disease. Heart Fail Rev.

[6] Bachar GN, Dicker D, Kornowski R, Atar E (2012). Epicardial adipose tissue as a predictor of coronary artery disease in asymptomatic subjects. Am J Cardiol.

[7] Sakamoto A, Ishizaka N, Imai Y, Ando J, Nagai R, Komuro I (2014). Association of serum IgG4 and soluble interleukin-2 receptor levels with epicardial adipose tissue and coronary artery calcification. Clin Chim Acta Int J Clin Chem.

[8] Beiert T, Knappe V, Tiyerili V, Stockigt F, Effelsberg V, Linhart M, Steinmetz M, Klein S, Schierwagen R, Trebicka J (2018). Chronic lower-dose relaxin administration protects from arrhythmia in experimental myocardial infarction due to anti-inflammatory and anti-fibrotic properties. Int J Cardiol.

[9] Horckmans M, Bianchini M, Santovito D, Megens RTA, Springael JY, Negri I, Vacca M, Di Eusanio M, Moschetta A, Weber C (2018). Pericardial adipose tissue regulates granulopoiesis, fibrosis, and cardiac function after myocardial infarction. Circulation.

[10] Park JS, Ahn SG, Hwang JW, Lim HS, Choi BJ, Choi SY, Yoon MH, Hwang GS, Tahk SJ, Shin JH (2010). Impact of body mass index on the relationship of epicardial adipose tissue to metabolic syndrome and coronary artery disease in an Asian population. Cardiovasc Diabetol.

[11] Ojha S, Fainberg HP, Wilson V, Pelella G, Castellanos M, May ST, Lotto AA, Sacks H, Symonds ME, Budge H (2016). Gene pathway development in human epicardial adipose tissue during early life. JCI Insight.

[12] Chechi K, Voisine P, Mathieu P, Laplante M, Bonnet S, Picard F, Joubert P, Richard D (2017). Functional characterization of the Ucp1-associated oxidative phenotype of human epicardial adipose tissue. Sci Rep.

[13] Chechi K, Vijay J, Voisine P, Mathieu P, Bosse Y, Tchernof A, Grundberg E, Richard D (2019). UCP1 expression-associated gene signatures of human epicardial adipose tissue. JCI Insight.

[14] Zangi L, Oliveira MS, Ye LY, Ma Q, Sultana N, Hadas Y, Chepurko E, Spater D, Zhou B, Chew WL (2017). Insulin-like growth factor 1 receptor-dependent pathway drives epicardial adipose tissue formation after myocardial injury. Circulation.

[15] Ritchie ME, Phipson B, Wu D, Hu Y, Law CW, Shi W, Smyth GK (2015). limma powers differential expression analyses for RNA-sequencing and microarray studies. Nucleic Acids Res.

[16] Newman AM, Liu CL, Green MR, Gentles AJ, Feng W, Xu Y, Hoang CD, Diehn M, Alizadeh AA (2015). Robust enumeration of cell subsets from tissue expression profiles. Nat Methods.

[17] Zhou Y, Zhou B, Pache L, Chang M, Khodabakhshi AH, Tanaseichuk O, Benner C, Chanda SK (2019). Metascape provides a biologist-oriented resource for the analysis of systems-level datasets. Nat Commun.

[18] Subramanian A, Tamayo P, Mootha VK, Mukherjee S, Ebert BL, Gillette MA, Paulovich A, Pomeroy SL, Golub TR, Lander ES (2005). Gene set enrichment analysis: a knowledge-based approach for interpreting genome-wide expression profiles. Proc Natl Acad Sci USA.

[19] Jeggari A, Marks DS, Larsson E (2012). miRcode: a map of putative microRNA target sites in the long non-coding transcriptome. Bioinformatics (Oxford, England).

[20] Wong N, Wang X (2015). miRDB: an online resource for microRNA target prediction and functional annotations. Nucleic Acids Res.

[21] Chou CH, Shrestha S, Yang CD, Chang NW, Lin YL, Liao KW, Huang WC, Sun TH, Tu SJ, Lee WH (2018). miRTarBase update 2018: a resource for experimentally validated microRNA-target interactions. Nucleic Acids Res.

[22] Agarwal V, Bell GW, Nam JW, Bartel DP (2015). Predicting effective microRNA target sites in mammalian mRNAs. eLife.

[23] Braschi B, Denny P, Gray K, Jones T, Seal R, Tweedie S, Yates B, Bruford E (2019). Genenames.org: the HGNC and VGNC resources in 2019. Nucleic Acids Res.

[24] Hanzelmann S, Castelo R, Guinney J (2013). GSVA: gene set variation analysis for microarray and RNA-seq data. BMC Bioinform.

[25] Tay Y, Rinn J, Pandolfi PP (2014). The multilayered complexity of ceRNA crosstalk and competition. Nature.

[26] An Y, Furber KL, Ji S (2017). Pseudogenes regulate parental gene expression via ceRNA network. J Cell Mol Med.

[27] Belinky F, Nativ N, Stelzer G, Zimmerman S, Iny Stein T, Safran M, Lancet D (2015). PathCards: multi-source consolidation of human biological pathways. Database.

[28] Weeke P, Muhammad R, Delaney JT, Shaffer C, Mosley JD, Blair M, Short L, Stubblefield T, Roden DM, Darbar D (2014). Whole-exome sequencing in familial atrial fibrillation. Eur Heart J.

[29] He Z, Dang J, Song A, Cui X, Ma Z, Zhang Z (2019). Identification of LINC01234 and MIR210HG as novel prognostic signature for colorectal adenocarcinoma. J Cell Physiol.

[30] Ruan Z, Xu Z, Li Z, Lv Y (2019). Integral analyses of survival-related long non-coding RNA MIR210HG and its prognostic role in colon cancer. Oncol Lett.

[31] Kang X, Kong F, Huang K, Li L, Li Z, Wang X, Zhang W, Wu X (2019). LncRNA MIR210HG promotes proliferation and invasion of non-small cell lung cancer by upregulating methylation of CACNA2D2 promoter via binding to DNMT1. OncoTargets Ther.

[32] Min W, Dai D, Wang J, Zhang D, Zhang Y, Han G, Zhang L, Chen C, Li X, Li Y (2016). Long noncoding RNA miR210HG as a potential biomarker for the diagnosis of glioma. PLoS ONE.

[33] Li XY, Zhou LY, Luo H, Zhu Q, Zuo L, Liu GY, Feng C, Zhao JY, Zhang YY, Li X (2019). The long noncoding RNA MIR210HG promotes tumor metastasis by acting as a ceRNA of miR-1226-3p to regulate mucin-1c expression in invasive breast cancer. Aging.

[34] Wang Y, Li W, Chen X, Li Y, Wen P, Xu F (2019). MIR210HG predicts poor prognosis and functions as an oncogenic lncRNA in hepatocellular carcinoma. Biomed Pharmacother Biomed Pharmacother.

[35] Lin J, Zhang X, Xue C, Zhang H, Shashaty MG, Gosai SJ, Meyer N, Grazioli A, Hinkle C, Caughey J (2015). The long noncoding RNA landscape in hypoxic and inflammatory renal epithelial injury. Am J Physiol Renal Physiol.

[36] Voellenkle C, Garcia-Manteiga JM, Pedrotti S, Perfetti A, De Toma I, Da Silva D, Maimone B, Greco S, Fasanaro P, Creo P (2016). Implication of Long noncoding RNAs in the endothelial cell response to hypoxia revealed by RNA-sequencing. Sci Rep.

[37] Sandbothe M, Buurman R, Reich N, Greiwe L, Vajen B, Gurlevik E, Schaffer V, Eilers M, Kuhnel F, Vaquero A (2017). The microRNA-449 family inhibits TGF-beta-mediated liver cancer cell migration by targeting SOX4. J Hepatol.

[38] Wu Z, Wang H, Fang S, Xu C (2015). MiR-449c inhibits gastric carcinoma growth. Life Sci.

[39] Wu J, Bao J, Kim M, Yuan S, Tang C, Zheng H, Mastick GS, Xu C, Yan W (2014). Two miRNA clusters, miR-34b/c and miR-449, are essential for normal brain development, motile ciliogenesis, and spermatogenesis. Proc Natl Acad Sci USA.

[40] Xu R, Zhao M, Yang Y, Huang Z, Shi C, Hou X, Zhao Y, Chen B, Xiao Z, Liu J (2017). MicroRNA-449c-5p inhibits osteogenic differentiation of human VICs through Smad4-mediated pathway. Sci Rep.

[41] Ahmadian M, Suh JM, Hah N, Liddle C, Atkins AR, Downes M, Evans RM (2013). PPARgamma signaling and metabolism: the good, the bad and the future. Nat Med.

[42] Fang H, Judd RL (2018). Adiponectin regulation and function. Compr Physiol.

[43] Delerive P, Fruchart JC, Staels B (2001). Peroxisome proliferator-activated receptors in inflammation control. J Endocrinol.

[44] Palomer X, Salvado L, Barroso E, Vazquez-Carrera M (2013). An overview of the crosstalk between inflammatory processes and metabolic dysregulation during diabetic cardiomyopathy. Int J Cardiol.

[45] Jia G, Hill MA, Sowers JR (2018). Diabetic cardiomyopathy: an update of mechanisms contributing to this clinical entity. Circ Res.

[46] Wang YH, Zhang YG (2017). Poly (I:C) alleviates obesity related pro-inflammatory status and promotes glucose homeostasis. Cytokine.

[47] Chattopadhyay S, Sen GC (2017). RIG-I-like receptor-induced IRF3 mediated pathway of apoptosis (RIPA): a new antiviral pathway. Protein Cell.

[48] Renovato-Martins M, Matheus ME, de Andrade IR, Moraes JA, da Silva SV, Citelli Dos Reis M, de Souza AA, da Silva CC, Bouskela E, Barja-Fidalgo C (2017). Microparticles derived from obese adipose tissue elicit a pro-inflammatory phenotype of CD16(+), CCR5(+) and TLR8(+) monocytes. Biochim Biophys Acta.

[49] Wolf Y, Boura-Halfon S, Cortese N, Haimon Z, Sar Shalom H, Kuperman Y, Kalchenko V, Brandis A, David E, Segal-Hayoun Y (2017). Brown-adipose-tissue macrophages control tissue innervation and homeostatic energy expenditure. Nat Immunol.

[50] Pirzgalska RM, Seixas E, Seidman JS, Link VM, Sanchez NM, Mahu I, Mendes R, Gres V, Kubasova N, Morris I (2017). Sympathetic neuron-associated macrophages contribute to obesity by importing and metabolizing norepinephrine. Nat Med.

[51] Barrett T, Wilhite SE, Ledoux P, Evangelista C, Kim IF, Tomashevsky M, Marshall KA, Phillippy KH, Sherman PM, Holko M (2013). NCBI GEO: archive for functional genomics data sets–update. Nucleic Acids Res.

